# Linking structural and compositional changes in archaeological human bone collagen: an FTIR-ATR approach

**DOI:** 10.1038/s41598-020-74993-y

**Published:** 2020-10-21

**Authors:** Antonio Martínez Cortizas, Olalla López-Costas

**Affiliations:** 1 EcoPast, Faculty of Biology, Campus Vida, Universidade de Santiago de Compostela, 15782 Santiago de Compostela, Spain; 2 Archaeological Research Laboratory, Stockholm University, Wallenberglaboratoriet, 10691 Stockholm, Sweden; 3 Laboratory of Anthropology, Department of Legal Medicine, Toxicology and Physical Anthropology, Faculty of Medicine, Universidad de Granada, 18012 Granada, Spain

**Keywords:** Biogeochemistry, Biogeochemistry

## Abstract

Collagen is the main structural and most abundant protein in the human body, and it is routinely extracted and analysed in scientific archaeology. Its degree of preservation is, therefore, crucial and several approaches are used to determine it. Spectroscopic techniques provide a cost-effective, non-destructive method to investigate the molecular structure, especially when combined with multivariate statistics (chemometric approach). In this study, we used FTIR-ATR spectroscopy to characterise collagen extracted from skeletons recovered from necropoleis in NW Spain spanning from the Bronze Age to eighteenth century AD. Principal components analysis was performed on a selection of bands and structural equation models (SEM) were developed to relate the collagen quality indicators to collagen structural change. Four principal components represented: (i) Cp1, transformations of the backbone protein with a residual increase in proteoglycans; (ii) Cp2, protein transformations not accompanied by changes in proteoglycans abundance; (iii) Cp3, variations in aliphatic side chains and (iv) Cp4, absorption of the OH of carbohydrates and amide. Highly explanatory SEM models were obtained for the traditional collagen quality indicators (collagen yield, C, N, C:N), but no relationship was found between quality and δ^13^C and δ^15^N ratios. The observed decrease in C and N content and increase in C:N ratios is controlled by the degradation of protein backbone components and the relative preservation of carbon-rich compounds, proteoglycans and, to a lesser extent, aliphatic moieties. Our results suggest that FTIR-ATR is an ideal technique for collagen characterization/pre-screening for palaeodiet, mobility and radiocarbon research.

## Introduction

Skeletal collagen is one of the most abundant proteins in vertebrate organisms, formed by a complex structure of fibres and microfibers that connect in a twisted, rope-like assembly^1,2^. Collagen 3D structure has been the focus of numerous studies in medical and biological sciences e.g.^3–6^ and special attention has been paid to the changes that occur at structural level since they can affect normal molecule functions in body e.g. sustention, connection, etc.^7^. Orthopaedic investigations have focused upon the degradation of human cartilage/bone, particularly the development of degenerative changes that result in osteoarthritis and cause modifications of the 3D structure, which occur in parallel with the advance of the disease see for example^8,9^.

Due to its abundance and strength in skeleton, it is possible to find collagen (mainly Type I) molecules in a human body several centuries after death and even after millions of years in fossil animals^10^. Archaeology, forensic science and physical anthropology routinely analyse extracted collagen to understand the *pre-mortem* features of the deceased (e.g. diet and mobility with stable isotopes, animal species through ZooMS) and to use for radiocarbon dating^11–15^. Most of these analyses are based on isotopic composition fractionation with strict control at elemental composition level to discard unsuitable samples. Common concerns with the extraction of collagen are the presence of exogenous substances (e.g. humid acids) and the loss of integrity of the collagen molecule. The first concern has been alleviated by the improvement of extraction methods to provide an improved level of certainty about the elimination of non-collagen substances^16^. Efforts to understand extracted collagen integrity/quality have mainly focused on applying elemental composition (i.e. C, N and C:N) cut-points, as described in^17^, to address the second concern.

In contrast to the in vivo molecule, archaeological collagen degradation models are complex because they need to consider changes that occurred during *post-mortem*. Some authors have used collagen quality^18,19^ or modelled linear structure^20^ as an indicator of bone degradation with time, and, in contrast to medical sciences, less attention has been paid to structural changes. Even now, the mechanisms and processes that influence the degradation of collagen extracted from archaeological bone samples are still poorly understood. To redress this, it is necessary to unravel the changes at structural level to achieve a good understanding of archaeological collagen preservation.

Despite its potential, few studies have used spectroscopic techniques to determine collagen preservation in archaeological bone^21–25^. Fourier Transform Infrared (FTIR) spectroscopy has been regarded as a suitable method to explore the structure of collagen^26–32^, by relating FTIR absorption bands (of the amide I, II and III) to specific chemical bonds and secondary structural features (α-helix, β-sheets, β-turns and random coils), even in the most recent investigations^32^. But, as early as the mid-twentieth century, there was a fundamental change in the comprehension of the collagen structure led by X-ray diffraction investigations, which showed that the traditional model was incorrect and the polyproline II (PPII) model was introduced and backed by later investigations, becoming the accepted model^4,33–36^. Although FTIR does not provide the same level of detail of the molecular composition compared with X-ray, Nuclear Magnetic Resonance (NMR) or Pyrolysis GC–MS, it can provide nonetheless valuable insights about the structure of complex molecules such as proteins^37^.

FTIR has many advantages when compared with the conventional methods commonly used to study collagen. It is a quick, cost-effective and non-invasive method^21,26^. Most studies that have used FTIR on ancient skeletons focus on the characterisation of the bone mineral component among others^38–41^ or on taphonomic processes such as cremation^42–44^. The collagenous portion of bone has been analysed with relatively less frequency using FTIR^15,22,45–47^, and Raman spectroscopy^21,48–50^. Studies of bulk bone have also demonstrated that it is difficult to detect collagen content in poorly preserved bones^47^, whereas extracted archaeological collagen has only been directly analysed in few studies^24,25,48^. Therefore, previous research focused upon establishing criteria or parameters for collagen preservation screening while the changes in the structure of the molecule have received much less attention.

The objective of our study is to characterise collagen extracted from archaeological human bone of different age, funerary context and burial environment, using FTIR-ATR in the mid infrared region (4000–400 cm^−1^). By using a combination of principal components analysis (PCA) and partial least squares-structural equation modelling (PLS-SEM), we (i) discuss the possible mechanisms of archaeological bone collagen structural transformation, (ii) the potential of FTIR-ATR to predict collagen quality indicators (i.e. C, N, C:N, collagen yield) and (iii) whether collagen quality affects its isotopic (δ^13^C and δ ^15^N) composition, which is key for the study of human palaeodiet and radiocarbon dating.

## Results

### Collagen properties

Of the fifty samples analysed, collagen yield ranged between 25% (similar to intact bone) and 2% (above the proposed limit of 1%^17^), whereas the C:N ratio was between 3.18 and 3.57. Carbon and nitrogen contents showed a larger range (C: 44.3–17.9%; N: 6.1–16.1%). None of the samples analysed in this study exceeded the C and N values of fresh collagen (43% and 16%, respectively^17,19^) by more than 3%. Eight and twelve samples provided percentage C and N values below 80% of those of fresh collagen respectively and two samples (424 and 705) were below 50%. Only one sample (424) showed a C:N ratio (3.57) slightly above the range (3.02–3.56) proposed as representative for well-preserved collagen^17^.

A wide distribution of isotopic results has been found in this study, especially for δ^13^C, which is interpreted as the result of palaeodietary preferences. For example, the observed differences in δ^13^C can be related to geographical location, whether coastal or inland, and δ^13^C was found to be influenced by the consumption of marine resources. Preference for the use of C_4_ plants in human and domestic animal diet and a strong reliance on seafood and fish—on the coast—occurs in North-Western Spain^51^. Historical and archaeological data agree with the obtained isotopic signatures and were discussed in detail for the analysed populations^52^.

For the samples used in this study, collagen yield shows significant, although low, correlations only with C, N and the C:N ratio (r 0.44, 0.49 and − 0.48, respectively; P < 0.01). Carbon and nitrogen contents are highly correlated (r 0.99; P < 0.01) with each other and are negatively correlated with the C:N ratio (− 0.76 and − 0.79, respectively; P < 0.01). Collagen compositional properties are not significantly correlated with the isotope ratios. Despite this, both isotope ratios are moderately correlated (r 0.55; P < 0.01), caused by the input of marine resources influencing some of the samples see^51^.

### Collagen FTIR-ATR spectra

**Figure 1:**
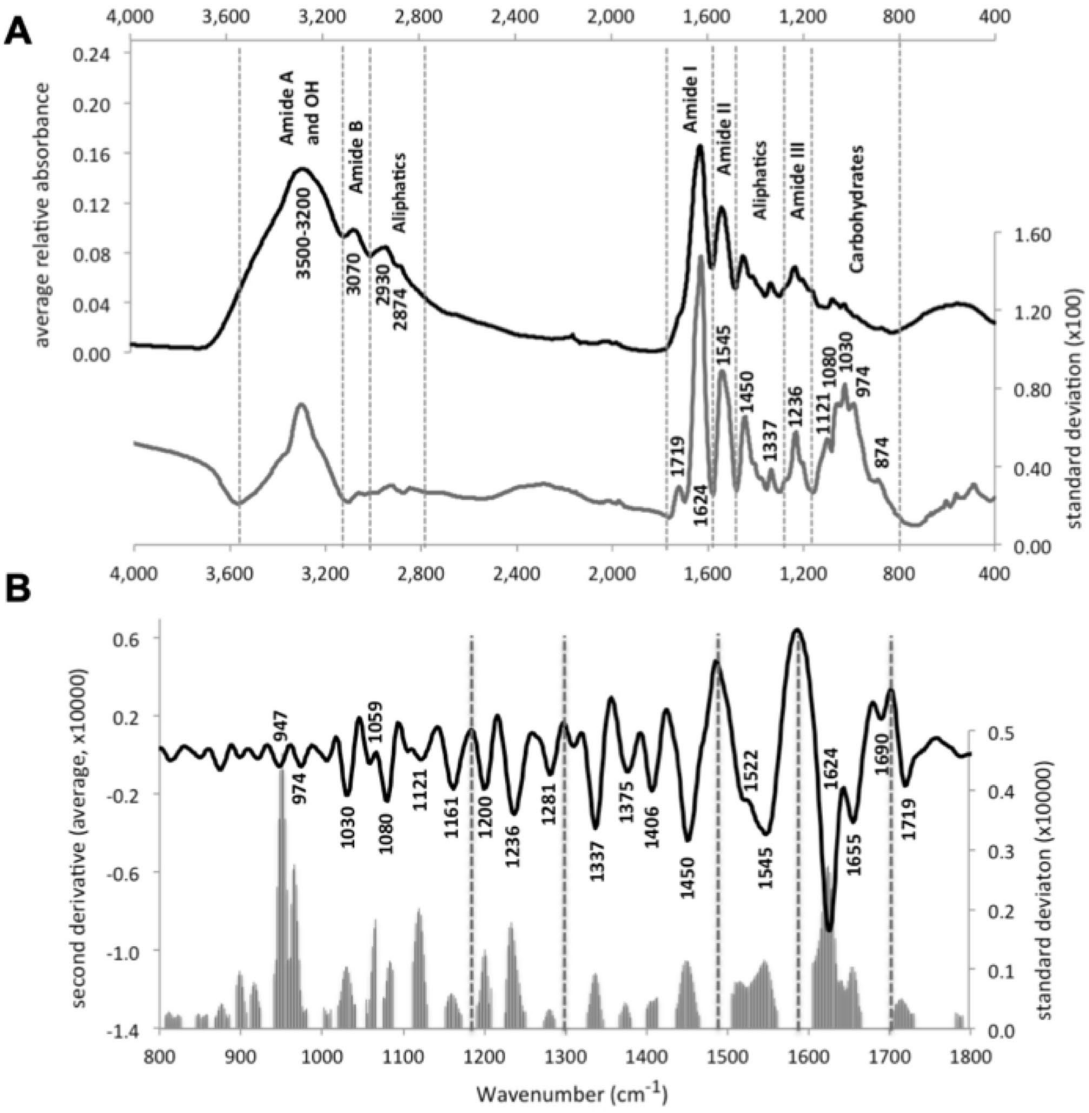
(**A**) Average (black line) and standard deviation (grey line) mid infrared FTIR-ATR spectra of the whole set of collagen samples analysed in this study. (**B**) Average spectrum of the second derivative spectra of the analysed samples in the 1800–800 cm^−1^ region; grey bars correspond to the standard deviation of the main absorptions. Vertical dashed lines separate the main collagen spectral regions (see “Collagen FTIR-ATR spectra” section).

The average spectrum of the samples shows the characteristic band distribution of collagen, with high absorbance in the regions 1500–1700 cm^−1^ and 2800–3500 cm^−1^, moderate absorbance at 1300–1500 cm^−1^ and relatively low average absorbance at 800–1200 cm^−1^ (Fig. 1a). The standard deviation spectrum is similar to the average one but shows a relatively large variation between samples in the region 800–1200 cm^−1^, despite its low average absorbance (Fig. 1a); whereas the 2800–3500 cm^−1^ region only presents a peak around 3300 cm^−1^.

The most relevant peaks obtained from the second derivative spectra, in the region 800–1800 cm^−1^, are shown in Fig. 1b. Assignment of the selected bands can be found in SI_Table 2. All spectra presented absorptions at 897, 918, 947, 974, 1030, 1059, 1080 and 1121 cm^−1^ that are characteristic of carbohydrate moieties (CO st and COC st); 1236 cm^−1^, of the amide III (CN st and NH d); 1337 and 1450 cm^−1^, attributable to methylene (CH_2_ d and CH_3_ d; hereon named as aliphatic) absorptions; 1545, 1624 and 1719 cm^−1^, due to amide II (CN st and NH bd) and amide I (mostly C = 0 st), respectively; 2874 and 2930 cm^−1^ assigned to aliphatics (CH st and CH_3_ st); 3070 cm^−1^, of the amide B (NH st); and a broad band 3500–3300 cm^−1^ related to amide A (NH st) and OH vibrations. Absorptions at 947, 974 and 1624 cm^−1^ showed the largest variability; while absorptions at 1030, 1059, 1080, 1121, 1200, 1236, 1337, 1450, 1545 and 1655 cm^−1^ displayed moderate variability (Fig. 1b).

### Main spectroscopic signals of collagen

We selected 24 bands, which are representative of the different spectral regions of the type I collagen spectrum (carbohydrates, amide III, miscellaneous—mainly aliphatics—region, amide II, amide I, aliphatics, amide B, amide A/OH; for a definition of these regions see for example^27,31^), to perform the PCA. Four principal components accounted for 95.5% of the variance (Table 1). The first component, Cp1, explains 45.5% of the total variance and it is characterised by large positive loadings (0.73–0.94) of absorptions of carbohydrates (i.e. collagen proteoglycans) and large negative loadings (− 0.86 to − 0.72) of absorptions of the amides (I, II and III) and the miscellaneous region (Table 1).

**Table 1:** Factor loadings of the IR band of the extracted components.

WN cm^−1^	Cp1	Cp2	Cp3	Cp4
897	**0.93**	0.33	0.07	− 0.05
918	**0.90**	0.38	0.12	0.06
947	**0.94**	0.29	0.09	0.01
974	**0.91**	0.29	0.10	0.22
1030	**0.73**	0.22	0.34	0.50
1059	**0.88**	0.21	0.34	0.23
1080	**0.91**	0.31	0.25	0.00
1121	**0.91**	0.33	0.14	− 0.07
1236	**− 0.75**	0.64	0.06	0.09
1337	**− 0.72**	0.62	0.16	0.08
1450	**− 0.72**	0.58	0.19	0.21
1522	**− 0.86**	0.41	0.25	0.15
1545	**− 0.80**	0.52	0.23	− 0.04
1655	**− 0.76**	0.49	0.19	− 0.11
1200	− 0.28	**0.93**	− 0.03	0.04
1624	− 0.59	**0.67**	0.37	0.07
1690	− 0.35	**0.87**	0.05	0.00
1719	0.09	**0.84**	− 0.33	0.02
2874	0.33	**0.76**	− 0.53	0.07
2930	0.02	**0.88**	− 0.44	0.13
2982	0.16	**0.89**	− 0.40	0.06
3070	0.20	**0.91**	− 0.30	0.07
3320	0.22	**0.73**	0.21	− 0.60
3458	0.46	0.58	0.15	− **0.63**
Eigv	10.9	9.2	1.6	1.2
Var	45.4	38.2	6.7	5.2

WN: wavenumber; Eigv: eigenvalue; Var: proportion of variance. The largest loading for each absorption band is in bold.

The second component, Cp2, explains 38.3% of the total variance. Amide (I, II, III, A and B) and aliphatic (CH_2_ and CH_3_) absorptions show large positive loadings (Table 1). Of the collagen absorption bands, Cp2 accounts for a large percentage of the 1690 cm^−1^ (76%) and a moderate percentage of 1624 cm^−1^ (45%) variance of amide I, and 1200 cm^−1^ (86%) of amide III. It also contains a low (24%) percentage of the variance the 1655 cm^−1^ absorption.

Components Cp3 and Cp4 account for a minor part of the total variance, 6.7 and 5.2%, respectively (Table 1). Absorptions related to aliphatics (2874, 2930 and 2982 cm^−1^) have the largest (albeit moderate to low) loadings in Cp3. While absorptions of the amide A/OH region (3320 and 3458 cm^−1^) and one of the carbohydrates bands (1030 cm^−1^) show moderate and opposed (negative and positive, respectively) loadings in Cp4 (Table 1).

Cp1 is highly correlated (P < 0.01) to the PGI, C and N content, and the C:N ratio (Table 2). Collagen yield is significantly correlated to Cp1 and Cp3, and the CI is negatively correlated with Cp3, although the correlation coefficients are low.

**Table 2:** Correlation between de extracted IR principal components.

	Cp1	Cp2	Cp3	Cp4
Coll_yield	0.42	0.21	0.41	− 0.22
CI	0.07	− 0.14	− 0.41	0.31
PGI	0.98	− 0.11	0.02	0.10
C	− 0.93	0.05	0.11	0.09
N	− 0.92	0.08	0.13	0.08
C:N	0.73	0.18	− 0.21	− 0.06
δ^13^C	0.00	− 0.15	− 0.09	0.17
δ ^15^N	0.23	− 0.07	− 0.17	0.18

Collagen yield (Coll_yield), IR indices (CI and PGI), elemental composition (C and N), C:N molar ratios and isotopic composition of the collagen.

### Modelling collagen quality and isotopic composition

The PCA results suggest that the spectroscopic nature of extracted bone collagen can provide insights into the main transformations of its composition and structure, which may be related to collagen preservation. To do so, we applied PLS-SEM modelling to determine (i) whether transformations of the collagen structure are coupled to changes in collagen quality (i.e. C, N, C:N, collagen yield), and (ii) if changes in collagen quality affect the isotopic (δ^13^C and δ ^15^N) composition. The model was initially designed with four predictor LV (amides, backbone lipids, side-chain lipids, and carbohydrates; SI_Figure 3), one primary response LV (collagen quality) and a secondary response LV (collagen isotopic composition; this one depending exclusively on collagen quality). As indicators, we used representative absorption bands for the predictor LV, analysed properties and indices (C, N, C:N, collagen yield, CI and PGI) and isotopic ratios (δ^13^C, δ ^15^N). Although the model predicted 92% of the collagen quality variance (SI_Figure 3), the lipids LV failed to pass the collinearity tests as it shared 88% of its variance with the amide LV and its total effect coefficient on collagen quality was very low (− 0.04). As a result, for the final model we merged this LV with the amides into one LV, named as “structural components”.

Of the 24 absorption bands used in the PCA, 17 met the criteria for good indicators (absolute value of the loading > 0.7, Table 3) and were kept in the model. It is worth remembering that the square of the outer loading accounts for the proportion of variance of the indicator that is captured by the LV in PLS-SEM reflective mode. The loadings of the FTIR absorbances, with only one exception (1200 cm^−1^, in the structural components LV), show that almost all their variance is captured by the modelled LV. Carbon, N, C:N and PGI also meet the criteria of good indicators of collagen quality, but collagen yield has a moderate loading and the CI a very low one (Table 3). While the PGI highly co-varies with the common collagen quality parameters and maybe a valid indicator, collagen yield and CI are not. For this specific model, collagen quality is thus related to the former. Collagen yield has some dependence on operator processing (inaccuracy in pipetting, filtering, etc.).

**Table 3:** Loadings of the indicators for the LV (predictors and responses) of the PLS-SEM model.

Indicator	LVsc	LVcb	LVsc	LVcq	LVis
897 cm^−1^		0.98			
918 cm^−1^		0.99			
947 cm^−1^		0.99			
974 cm^−1^		0.98			
1059 cm^−1^		0.95			
1080 cm^−1^		0.98			
1200 cm^−1^	0.78				
1236 cm^−1^	0.99				
1337 cm^−1^	0.97				
1450 cm^−1^	0.95				
1522 cm^−1^	0.96				
1545 cm^−1^	0.97				
1624 cm^−1^	0.94				
1655 cm^−1^	0.91				
2874 cm^−1^			0.97		
2930 cm^−1^			0.99		
2982 cm^−1^			0.99		
C				0.96	
N				0.97	
C:N				− 0.84	
Coll_yield				0.61	
CI				− 0.19	
PGI				− 0.92	
δ^13^C					0.68
δ ^15^N					0.98

LVsc: structural components, LVcb: carbohydrates, LVsc: side chains, LVcq: collagen quality, LVis: collagen isotopic composition.

**Figure 2:**
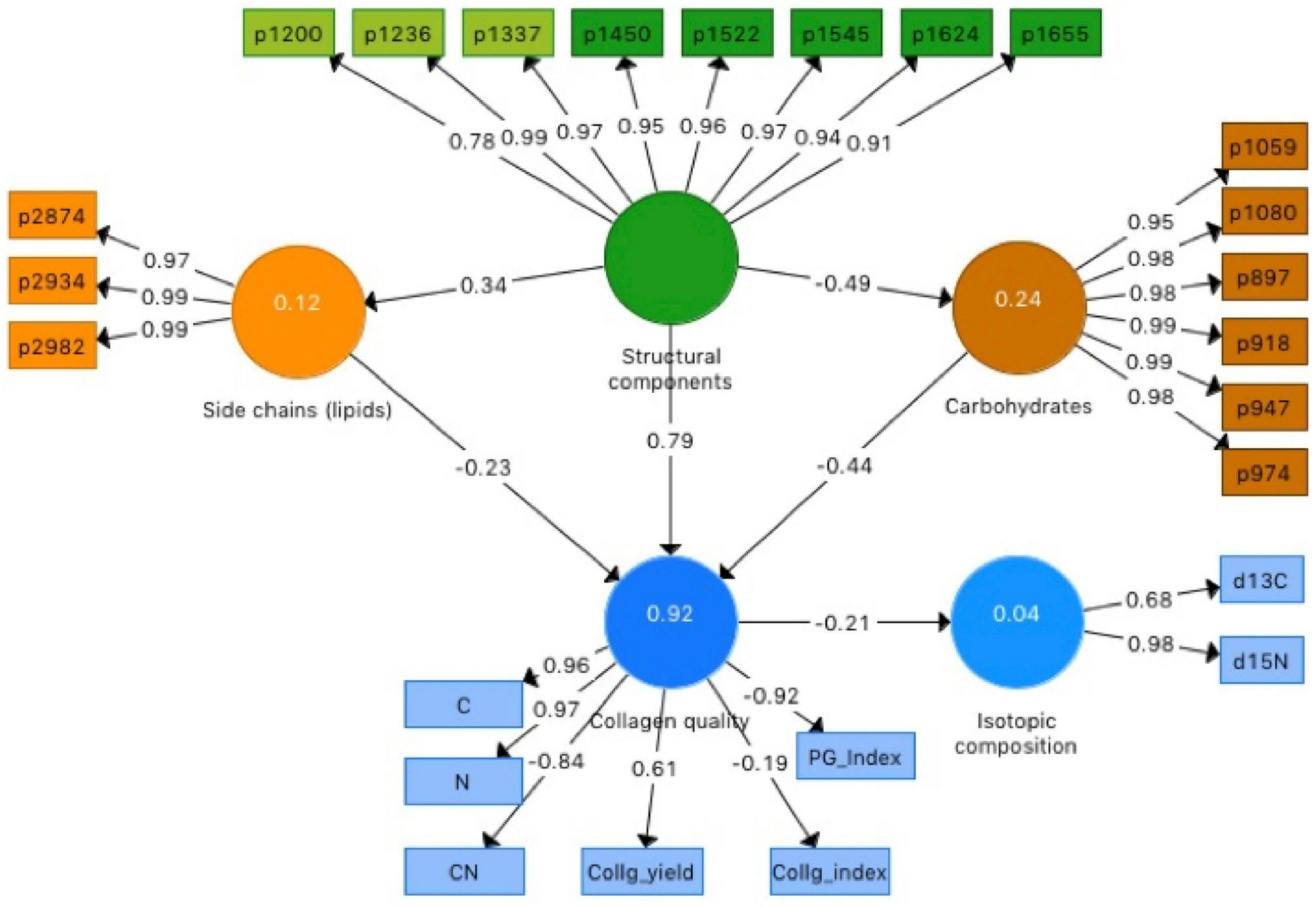
Total effects coefficients of the final PLS-structural equation model, including three predictor LVs (structural components, polysaccharides, side chain lipids), one primary response LV (collagen quality) and a secondary response LV (isotopic composition). Proxies of the predictor LVs are identified by the wavenumbers of the main absorptions of collagen components.

**Figure 3:**
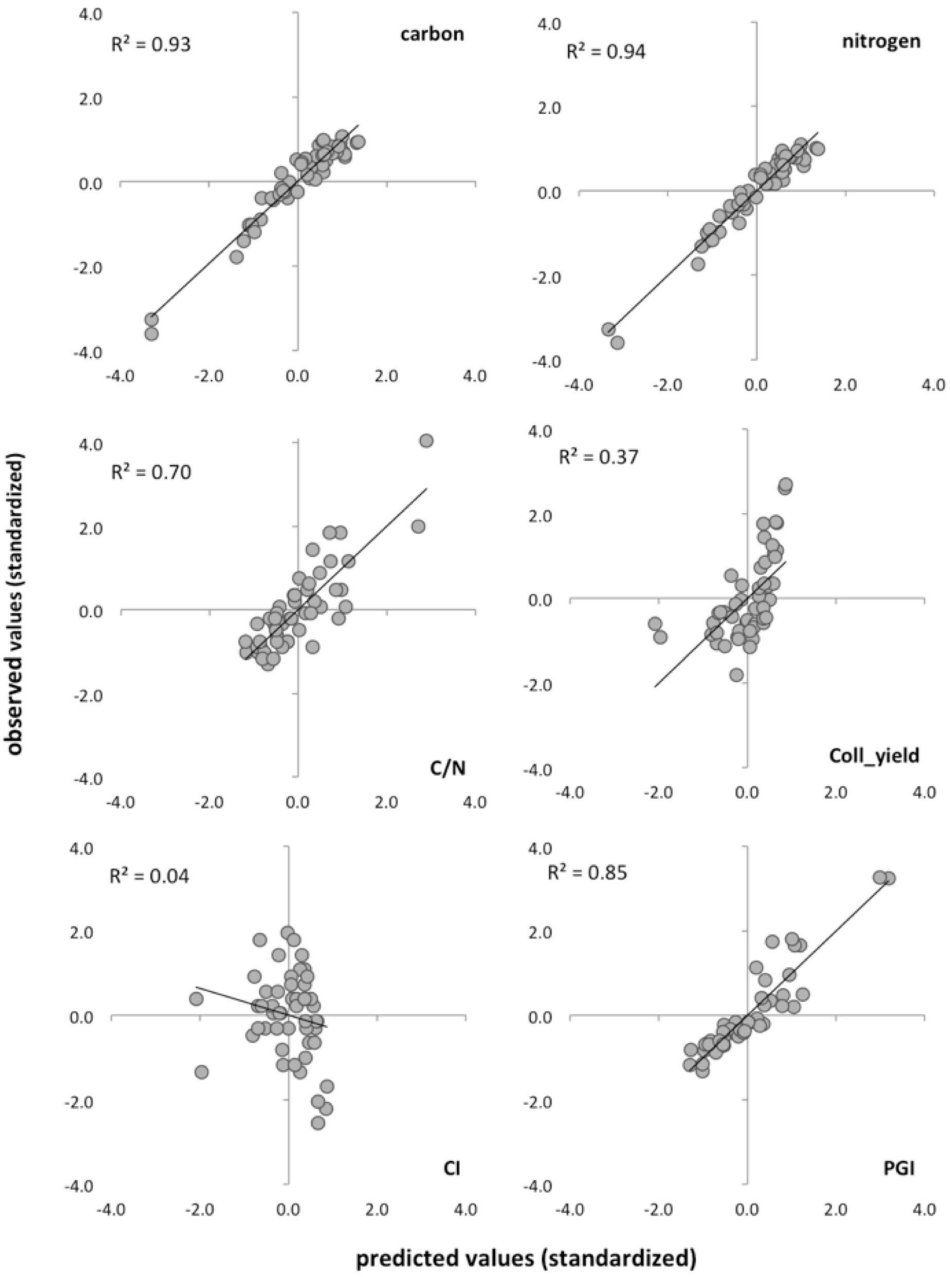
Correlation (coefficient of determination) between predicted (PLS-SEM model) and observed (standardized)-values for the main quality criteria indicators of collagen quality, plus de PGI and CI indices.

The total effects’ coefficients (Fig. 2) show that the structural components have the strongest, positive effect (0.79) on collagen quality, while carbohydrates and side-chain lipids have negative total effects (− 0.43 and − 0.22 respectively). The weight of the structural components on collagen quality is almost two and four times higher than the weights of the other two LVs. This simple PLS-SEM model explains 92% of the variation in collagen quality (Fig. 2), involving as much as 92–94% of the C and N, 85% of the PGI and 70% of the C:N variance. Figure 3 shows the relationship between observed and expected values for the collagen quality indicators obtained with the PLS-SEM model. Total C and N contents and the PGI are accurately estimated, C:N ratios also show a good albeit lower performance, estimation of collagen yield is moderate and that of the CI is not significant.

Additionally, at this level, collagen quality seems to have no significant effect on the isotopic composition: its total effect coefficient on the isotopic composition is low and the explained variance is almost negligible (4%).

## Discussion

The results of the PCA are in agreement with previous investigations that use FTIR spectra to provide additional insights on protein, in particular collagen, composition and structure^29,31,32,37,53–55^. Different collagen types can be identified/discriminated efficiently using absorbances from selected regions of the spectrum^27^.

In the samples analysed here, Cp1 and Cp2 seems to reflect a loss of protein backbone components. As most of the variation of the characteristic absorption of the aliphatic bonds (at 1337, 1450, 2874, 2934, and 2982 cm^−1^) are also contained in Cp1 and Cp2, and only a smaller proportion is captured by Cp3 (Table 2), it is likely that vibrations in the first two components are related to the methylene present in the backbone peptide structure whereas Cp3 may correspond to the aliphatic side chains. Cp4 seems to discriminate between the OH absorption of carbohydrates and that of the amide A.

**Figure 4:**
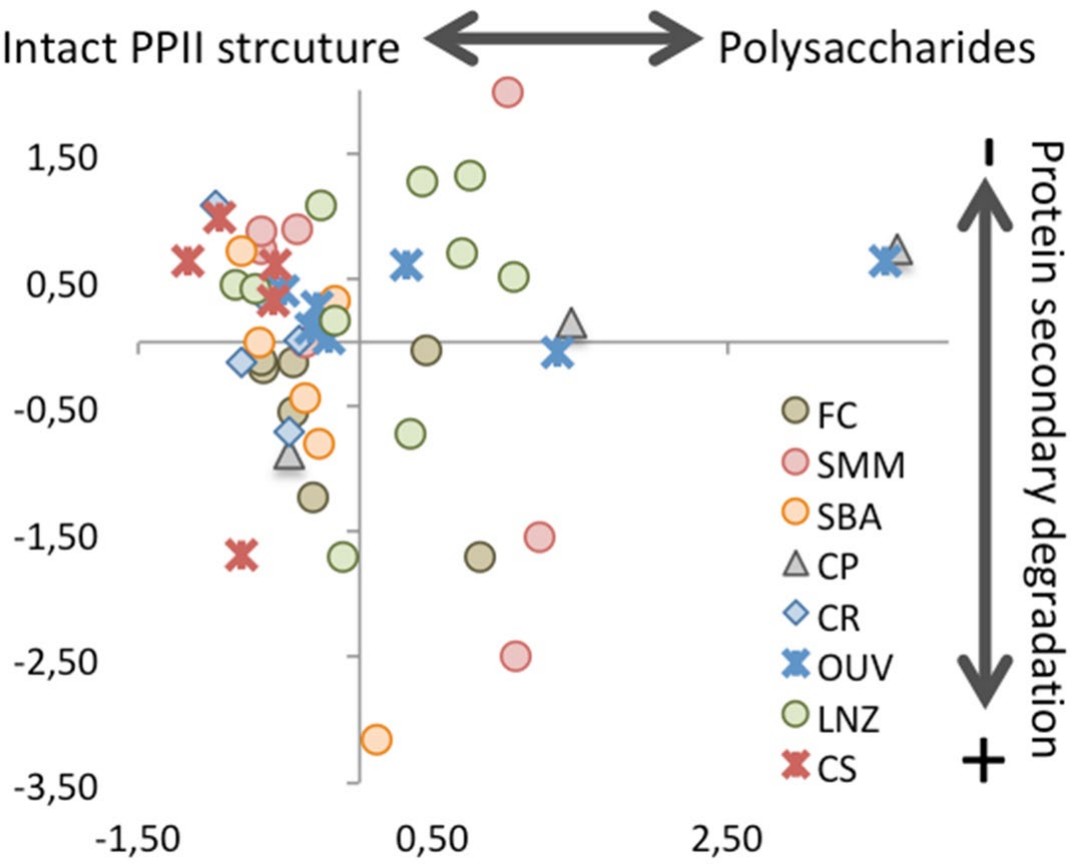
Cp1-Cp2 projection of the PCA samples scores. FC: mass grave from post-medieval times (seventeenth or eighteenth century AD); SMM: Santa María church (Pontevedra), medieval cemetery (thirteenth–seventeenth century AD); SBA: San Bartolomé medieval churchyard (thirteenth–fifteenth century AD); CP: Capela do Pilar, inhumations from a chapel of the Lugo Cathedral (eleventh–fourteenth century AD); CR: Rúa Real, post-Roman (fifth–seventh century AD) inhumation necropolis; OUV: Ouvigo, Early-medieval (tenth–twelfth century AD) cemetery with a minor phase of burials from the Roman period (second–third century AD); LNZ: A Lanzada, inhumations from Roman and post-Roman times (first–seventh century AD); CS: Cova do Santo (nineteenth–sixteenth century BC), Bronze Age human remains found at a cavern surface.

Figure 4 represents a projection of samples’ scores for Cp1 and Cp2. Most samples (28 out of 50) show negative Cp1 scores and positive or slightly negative Cp2 scores. These may represent collagen with a more intact, PPII-like, molecular structure. Twelve samples show positive Cp1 scores and positive or slightly negative Cp2 scores, suggesting some degree of collagen transformation not affecting the main protein backbone structures. Samples with positive Cp1 and negative Cp2 scores may correspond to those with more intense structural modifications. Collagen quality parameters (C, N and C:N) with the most pronounced departure from those of fresh collagen occur in the two samples with the largest Cp1 values (424 from Ouvigo and 705 from Capela do Pilar; Fig. 4). No evidence of soil contamination (i.e. humic acids) was detected. Our results are consistent with findings in a previous molecular study which used pyrolysis-GC–MS on 28 of the samples analysed here^16^. Although a detailed comparison with the molecular data cannot be done, there is an overall agreement in the classification of collagen as well or poorly preserved (20 samples out of 28).

The PLS-SEM model (Fig. 2) suggests that the more intact the collagen backbone structure (reflected by LVst), the higher collagen quality (higher C and N contents and, to some extent, collagen yield), while lower quality (higher C:N ratios and PGI values) is characterised by the relative abundance of carbohydrates (LVcb) and, to a limited extent, lipidic side chains (LVsc). Collagen transformation results in an overall decrease in C and N, and an increase in C:N ratios and the PGI. This points to selective bacterial degradation of the protein component (amides and backbone lipids) and the relative preservation of carbohydrates and lipidic side chains. In fact, the negative coefficient for the interaction between the structural components (LVst) and the carbohydrates (LVcb) accounts for the increase in carbohydrates as the protein component decreases, which is consistent with the results of the PCA. The ratio 1660/1690 cm^−1^, related to maturity of collagen cross-links^56^, is negatively correlated to collagen quality (LVcq; r − 0.77, P < 0.01) and positively correlated to carbohydrates (LVcb) and side-chain lipids (LVsc) (r 0.67 and 0.79, P < 0.01, respectively), also consistent with the PCA results. It has been proposed that the loss of spectral intensity of collagen backbone structures is most likely related to the fragmentation of the molecule due to bacterial preference for the relatively high-energy amide bonds^21^. Altogether, this reinforces the idea that the main collagen transformation in the samples analysed here is controlled by the degradation of the amide backbone structure. However, it is not possible to assess whether bacterial degradation occurred during body putrefaction or later soil contact.

Raman analysis of collagen has shown that decreasing yield is accompanied by disappearance of amide peaks but not necessarily of aliphatic (C-H) components, since poorly preserved collagen samples produced spectra with well-defined aliphatic peaks^21^. Another study found that changes in amino acid composition alone could not account for the elevated C:N ratios in low collagen bone from experimentally aged human bones^18^. Moreover, low-collagen samples are more likely to show elevated ratios than contaminated samples^17^. Our results are in line with these observations since the less intact collagen samples are enriched in C-rich compounds (carbohydrates from proteoglycans and side chain lipids) and thus the C:N is expected to increase as degradation progresses. Although the presence of small amounts of non-carbon and non-nitrogen rich contaminants, as detected in other studies^57^, cannot be dismissed, their quantity was not deemed large enough to produce a detectable signal in the spectra.

**Figure 5:**
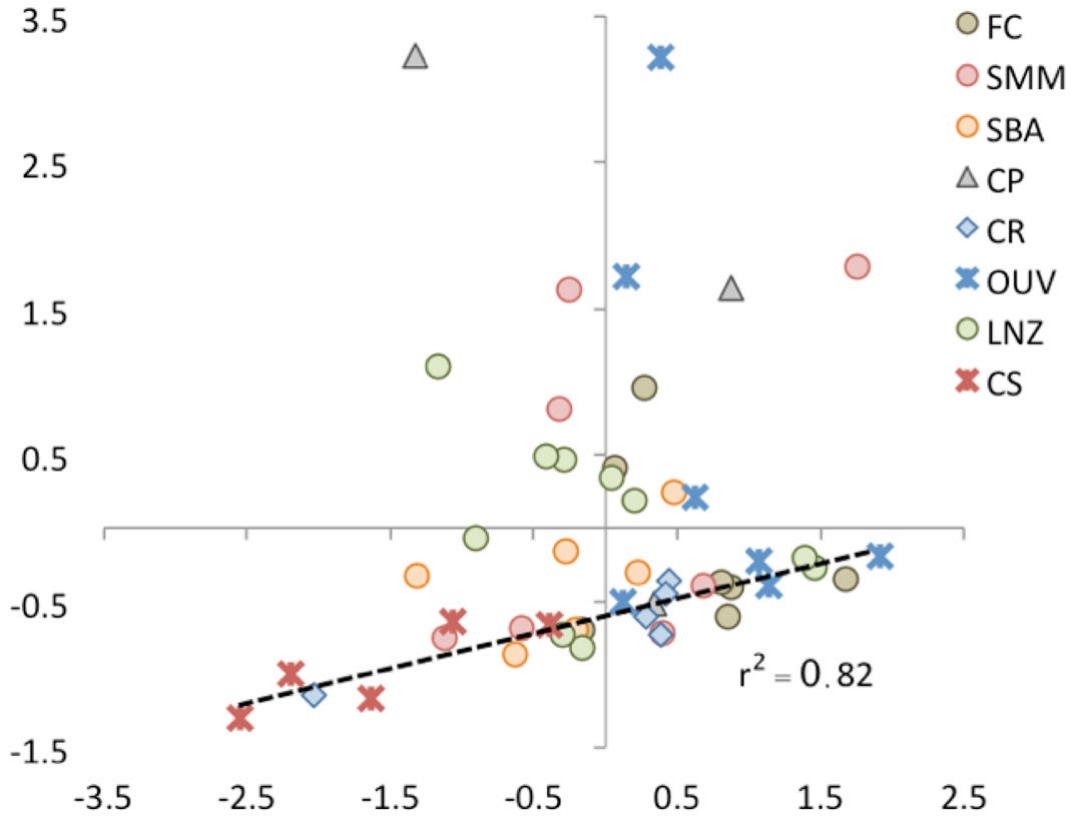
Correlation between the (standardized)-values of the PGI and CI indices. Samples showing good collagen integrity are highly correlated (those fitting the dashed line).

Another interesting feature is that the best-preserved samples characterised by negative Cp1 scores (Fig. 4) show a high correlation (r 0.91; P < 0.01) between the CI and the PGI (Fig. 5): the relative abundance of aliphatics and carbohydrates to the amide component tends to remain constant. In our opinion, this result has potential for the assessment of collagen transformation and integrity using FTIR-ATR; the larger the departure from the trend the more degraded the collagen structure.

The model also suggests that collagen quality (i.e. C, N, C:N and collagen yield) has no significant effect on the isotopic composition of the collagen. This is also consistent with the PCA and correlation results obtained here and in previous investigations, since no correlation was found between molecular indicators of collagen diagenesis and isotopic composition^16^. Other research also found that the isotopic values (δ^13^C and δ ^15^N) and C:N ratios of the insoluble fraction remained almost stable until collagen yield represented less than 1%^18^.

We performed ANOVA tests on the LV scores of the PLS-SEM model, using the necropoleis, archaeological period (Bronze Age to Modern period), burial environment (acidic or alkaline), sex (male or female), type of bone and age-at-death (< 19, 20–39, 40–59, > 60 estimated years old) as grouping variables. No significant differences were found for any of the LV scores (structural components, carbohydrates, side-chain lipids, and collagen quality) for archaeological period, type of bone, sex and age-at-death (SI_Table 3). Archaeological site and burial context presented significant differences for collagen quality (LVcq) and structural components (LVst) and carbohydrates (LVcb) for archaeological site only. Structural components (amides and backbone lipids) content was higher and carbohydrates content lower in Cova do Santo and Rúa Real compared to Capela do Pilar samples; the other necropoleis showing intermediate values between these two extremes. As a result, collagen quality was significantly higher in Cova do Santo and Rúa Real than in Capela do Pilar. In the latter case, the good macroscopic preservation of the skeletons does not agree with that suggested by the degree of integrity of the collagen structure.

As for the burial context, the alkaline environments (the cave on limestone and the palaeodunes with biogenic carbonates) showed better collagen preservation than the acidic ones as found in previous research e.g.^58,59^. Although not significant at P < 0.05, structural components and carbohydrates were higher and lower (P < 0.10) respectively in the alkaline environments. Thus, alkaline conditions seem to be the main reason for the good quality of the collagen of samples from Cova do Santo (limestone cave) and those of Rúa Real and A Lanzada (burials on palaeodunes). This is perhaps surprising given the sensitivity of collagen to hydrolysis under alkaline conditions^20^. The reasons for this apparent disagreement may be explained by (i) relatively low alkalinity in the burial contexts (pH < 9), the rate of collagen hydrolysis largely increasing above pH 11^20^; (ii) well-drained/aerated conditions predominate; (iii) low decomposition of collagen matrix preventing post-mortem alteration in bone mineral crystal^60,61^; and (iv) the dissolution of the bone mineral phase is retarded, limiting collagen exposition to enzymatic attack.

Recent research at A Lanzada concluded that the intensity of bone diagenesis was larger in burials in acidic soils than those on palaeodunes, regardless of the period (Roman or post-Roman)^59^. The confined environment of Cova do Santo cave could have had a larger effect than the high pH, as it was observed on research made in catacombs^62^. However, the particular mineral content of groundwater in this cave could also have promoted collagen preservation^63^. In our previous study of collagen molecular composition^16^, we identified a depolymerization process that differed depending on burial environment: acidic (soils/sediments) showing higher degree of depolymerization than alkaline (sand dunes and limestone cave) environments. Acidic conditions, which have been found to be the main cause of bioapatite alteration^41,59^ and promotion of collagen dissolution^64^, seem to be also important in the preservation of the protein structure—regardless of the chronological age. The oldest bones were the ones with the best preservation in our study. Finally, pH has been considered as part of “the site hydrology”—including also the mineral content of groundwater—a much more general factor that controls bone preservation^65^. In our study, well-drained sites (e.g. palaeodunes, such as the ones from Calle Real and A Lanzada) and places with constrained water movement (caves, as Cova do Santo) provided the best conditions for preservation. In both areas, groundwater is probably oversaturated for calcium phosphate, which would explain the good preservation of mineral and organic phases of the bone. The humidity of the soil can also promote bone degradation through microbial and fungal attack since alteration by microorganisms seems to dominate in temperate regions^63^^: p.114^. Humid conditions in NW Spain favour fungi in those soils neither well-drained nor anoxic. In addition, bones from Cova do Santo were exposed (not buried), which may have resulted in different *postmortem* changes^61,63^.

Despite these reservations, we conclude that there is no single factor to explain the changes in collagen structure. All necropoleis presented relatively large variations in their samples´ collagen structural components (Fig. 3); i.e. we found a range of preservation within populations rather than between populations of well/poorly preserved collagen. This may indicate that within any given geochemical environment conditions occurring at microscale may determine the intensity of degradation of collagen, an idea that has been suggested for the alteration of the mineral part of the bone^59,66–68^. Microorganism attack on bone is also a complex process^69^ with alterations caused by bacteria and fungi occurring on different scales and dependent on the *perimortem* and *postmortem* characteristics of the specific inhumation, which are difficult to fully appreciate in the current study. Raman studies also found spectral heterogeneity on bone crossed-sectioned surfaces, which was interpreted to indicate heterogeneous preservation of the collagen within a single bone^21^.

## Conclusions

Chemical transformation on archaeological human skeletons is a topic approached from different perspectives. Despite of this intense work, some authors have remarked upon the improvement of evaluating collagen preservation as a key factor to understand the interaction between bone and burial environment^65^. As far as we know, ours is the first study to analyse extracted collagen from human archaeological bone using FTIR-ATR, instead in bulk bone. Our findings indicate that there is a continuous change in C, N, and C:N ratios that is coupled to the integrity of the collagen structure: C and N decrease and C:N ratio increases as the protein structures are degraded and carbohydrates (and aliphatic side chains) are preserved, resulting in a relative increase in C-rich compounds. This transformation may explain why the discarded collagen samples in isotopic studies used to have high C:N values. However, the observed structural and compositional changes did not affect, in a significant way, the δ^13^C and δ^15^N values, thus supporting their use for palaeodiet reconstruction and radiocarbon dating. Additionally, we found that the carbohydrates/amide I index (PGI) is a potential reliable indicator of the compositional change of the collagen; the combination of the PGI with the CI may be of use to identify well-structured (i.e. preserved) collagen using FTIR-ATR. Thus, FTIR-ATR is an ideal technique for characterizing/pre-screening extracted collagen that is to be used for other destructive, more time consuming and expensive techniques in palaeodiet, mobility and radiocarbon research. For a full understanding of the link between structural and compositional changes in collagen, more research should be done for example by including samples not fulfilling all the “good-quality” criteria. There is the risk of inducing a bias in the results by analysing only those samples fulfilling the criteria^48,70^, as these are the ones expected to show less transformations of the molecule structure.

## Materials and methods

### Sample selection, collagen extraction and collagen properties

Collagen was obtained from fifty human skeletons recovered from eight necropoleis located in NW Iberia (SI_Figure 1, Table 1): (1) Cova do Santo (nineteenth–sixteenth century BC), where Bronze Age human remains found at a cavern surface provide one of the oldest examples of funerary deposits in NW Spain^71^; (2) A Lanzada (first–seventh century AD), a coastal, rural archaeological settlement with inhumations from Roman and post-Roman times^51,71,72^; (3) Rúa Real, a post-Roman (fifth–seventh century AD) inhumation necropolis located in the current city of A Coruña; (4) Ouvigo, an Early-medieval cemetery with a minor phase of burials from the Late Roman period (fourth–thirteenth century AD); (5) Capela do Pilar, inhumations from a chapel of the Lugo Cathedral (eleventh–fourteenth century AD); (6) San Bartolomé, medieval churchyard burials (thirteenth–fifteenth century AD); (7) Santa María church (Pontevedra), a medieval cemetery (thirteenth–seventeenth century AD) and (8) a mass grave from post-medieval times (seventeenth or eighteenth century AD)^16,52^. These necropoleis represent different archaeological/cultural periods (Bronze Age to post-Medieval) but also cover different geochemical environments (ranging from acidic soils, palaeodunes with biogenic carbonates to a cave formed in limestone) and different types of funerary contexts with human remains (SI_Table 1). The analyzed samples were selected according to bone surface preservation and available skeletal pieces, mainly ribs and long bones. Pathological bones were avoided. The individuals were estimated to be adults (18–60 years old) from both sexes (23 males, 20 females; and 7 undetermined). More archaeological, palaeodietary and osteological information about the necropoleis can be found elsewhere^51^. The climate of the area is temperate and moderately humid, providing good conditions for collagen preservation, with only slow losses expected to have taken place^17^.

The collagen extraction procedure followed^11^, with modifications by^73^. Small pieces of cortical bone (100–200 mg) were cleaned by removing 1–2 mm of the outer surface and demineralized in HCl (0.5 M) at low temperature (4 ºC) over approximately a week, in order to limit protein alteration. Samples were then heated (48 h at 70 ºC) in a weak (pH 3) HCl solution in order to gelatinize the collagen. The resulting solution was filtered (Ezee-filter™) and freeze-dried. Recent FTIR research on collagen type I^32^ has shown that heating between 20 and 80 ºC affects the relative intensity of some of the amide I and amide III vibrations. The intensity reduction/enhancement was lower than 5% for most of the bands and much of the change occurred between 40 and 50 ºC, stabilizing thereafter. Thus, the protocol we used to extract collagen was likely to produce a slight reduction in some bands absorbance. Since all samples were treated equally, this effect is not considered to have had a significant effect on the statistical associations and modeling.

Collagen properties (% C, % N, C:N ratio), often used to evaluate its degree of preservation, and stable isotope ratios (δ^13^C, δ ^15^N) were determined (in duplication) using an Europa 20–20 isotope ratio mass spectrometer coupled to a Sercon elemental analyzer, in the Department of Archaeology of the University of Reading (UK). Collagen yield was calculated as the wt% of collagen in archaeological bone. The results and discussion of these analyses have been described elsewhere^51,52,71,72^. All selected samples were considered to meet the criteria to be suitable for isotopic (δ ^13^C and δ ^15^N) study.

### Infrared measurements and peak selection, IR indices

FTIR spectra (4000–400 cm^−1^) were acquired at 4 cm^−1^ resolution by using a Gladi-ATR (Pike Technologies) spectrometer at the IR-Raman facility of the RIAIDT (Universidade de Santiago de Compostela, Spain). All spectra were background corrected and smoothed with the Savitzky–Golay filter. Both processes were computed into Resolutions Pro FTIR Software (Agilent Technologies, USA) (a figure with all 50 spectra can be found un supporting information, SI_Figure 2). For the sake of representation, and given all spectra showed the same vibrational features, the average spectrum and the standard deviation spectrum were computed. In this way the average spectrum provides an overall figure for the whole set of samples analysed, while the standard deviation spectrum enables to highlight which regions of the mid infrared spectrum showed the greatest variability between samples (that is, where most of the information on the differences between the samples is located).

Additionally, two indices, the collagen index (CI) and carbohydrate/amide I index (PGI)—similar to the proteoglycan/amide I index, previously proposed as markers of cartilage degeneration^8,74–76^, were calculated from the IR spectra to check their validity to determine collagen compositional change:

$$ {\text{CI}} = {\text{ collagen CH}}_{{2}} \left( {{\text{1338 cm}}^{{ - {1}}} } \right)/{\text{amide II }}\left( {{1490}{-}{159}0{\text{ cm}}^{{ - {1}}} } \right) $$

$$ {\text{PGI}} = {\text{ carbohydrate C}}{-}{\text{O }}\left( {{\text{985 to }}\; 1140\,{\text{ cm}}^{{ - {1}}} } \right)/{\text{amide I }}\left( {{159}0{\text{ to}}\;1720\,{\text{ cm}}^{{ - {1}}} } \right) $$

The second derivative of infrared spectra was used for a more detailed structural characterisation of the collagen^77,78^ of all samples. This is a highly suitable method for peak identification as it enhances sharp bands, allowing to search peaks that are barely visible in the raw spectra^27,79,80^, as well as providing information into the structure of proteins^31^. Peak selection was done by locating minima in the second derivative as described in^81^. When evaluating the position of the relevant peaks in the second derivative spectra, we allowed for a ± 4 cm^−1^ interval.

### Statistical methods

The amount of information contained in each IR spectrum is rather large and the identification of the spectral regions that play a decisive role in the differences between collagen samples becomes quite complex. We applied principal components analysis (PCA) to 24 characteristic collagen vibrations detected with the second derivative to determine the main spectroscopic signatures and their variation for the set of samples analysed. PCA analysis was carried out on correlation mode, with varimax rotation (i.e. maximizing the loadings of the variables), after all variables were standardized (Z-scores = (xi-avg)/std), xi being the absorbance value at any wavenumber, “avg” the average absorbance of the spectrum and “std” the standard deviation of the spectrum) to avoid scaling effects^82^.

With the insights gained in the PCA we developed a PLS-SEM model. This technique was chosen because, in comparison with other multivariate fitting/predicting techniques, it reduces the dimension of predicting variables (only a few latent variables—LV—are used), avoids multicollinearity (the LV are orthogonal), deals robustly with fat matrices (low-moderate number of cases in relation to the number of variables) and enables to calculate direct and indirect effects^83^. In PLS-SEM, predictor LV are defined to maximize the explanation of the variance of the response LV^83^.

In our model, collagen components (amides, lipids, carbohydrates) and collagen quality were defined as latent variables (LV). As indicators of the latent variables we used the characteristic vibrations of the collagen components (see below) and C, N, C:N, collagen yield, the CI index and the PGI index for collagen quality. We aimed to test whether transformations of the collagen structure were coupled to changes in collagen quality (i.e. in C, N, C:N, collagen yield, CI and PGI). A second objective was to assess if changes in collagen quality affected the isotopic composition, so an additional latent variable was included, being δ^13^C and δ ^15^N its indicators. The model was performed in reflective mode (i.e. indicators as proxies of the latent variables) using the specific software for PLS-SEM modelling SmartPLS^84^.

## Supplementary information

Supplementary Information.

## References

[1] Boskey AL, Wright TM, Blank RD (1999). Collagen and bone strength. J. Bone Miner. Res..

[2] Fratzl, P. In *Collagen* (ed Fratzl, P.) 1–13 (Springer, Berlin, 2008).

[3] Dehring KA, Smukler AR, Roessler BJ, Morris MD (2006). correlating changes in collagen secondary structure with aging and defective type II collagen by Raman spectroscopy. Appl. Spectrosc..

[4] Shoulders MD, Raines RT (2009). Collagen structure and stability. Annu. Rev. Biochem..

[5] Mostaço-Guidolin LB (2013). Collagen morphology and texture analysis: From statistics to classification. Sci. Rep..

[6] Schrof S, Varga P, Galvis L, Raum K, Masic A (2014). 3D Raman mapping of the collagen fibril orientation in human osteonal lamellae. J. Struct. Biol..

[7] Viguet-Carrin S, Garnero P, Delmas PD (2006). The role of collagen in bone strength. Osteoporos. Int..

[8] West P, Torzilli P, Chen C, Lin P, Camacho N (2005). Fourier transform infrared imaging spectroscopy analysis of collagenase-induced cartilage degradation. J. Biomed. Opt..

[9] Wang X, Zhai M, Zhao Y, Yin J (2018). A review of articular cartilage and osteoarthritis studies by Fourier transform infrared spectroscopic imaging. Ann. Joint.

[10] Lee Y-C (2017). Evidence of preserved collagen in an Early Jurassic sauropodomorph dinosaur revealed by synchrotron FTIR microspectroscopy. Nat. Commun..

[11] Longin R (1971). New method of collagen extraction for radiocarbon dating. Nature.

[12] Ambrose SH, Krigbaum J (2003). Bone chemistry and bioarchaeology. J. Anthropol. Archaeol..

[13] 13Katzenberg, M. A. In *Biological Anthropology of the Human Skeleton* (eds M. Katzenberg, A. & Saunders, S. R.) 413–441 (Wiley-Liss, Hoboken, 2000).

[14] Fewlass H (2019). Pretreatment and gaseous radiocarbon dating of 40–100 mg archaeological bone. Sci. Rep..

[15] Pothier Bouchard G (2019). Portable FTIR for on-site screening of archaeological bone intended for ZooMS collagen fingerprint analysis. J. Archaeol. Sci. Rep..

[16] Kaal J, López-Costas O, Martínez A (2016). Diagenetic effects on pyrolysis fingerprints of extracted collagen in archaeological human bones from NW Spain, as determined by pyrolysis-GC-MS. J. Archaeol. Sci..

[17] Van Klinken GJ (1999). Bone collagen quality indicators for palaeodietary and radiocarbon measurements. J. Archaeol. Sci..

[18] Dobberstein RC (2009). Archaeological collagen: Why worry about collagen diagenesis?. Archaeol. Anthropol. Sci..

[19] Harbeck M, Grupe G (2009). Experimental chemical degradation compared to natural diagenetic alteration of collagen: Implications for collagen quality indicators for stable isotope analysis. Archaeol. Anthropol. Sci..

[20] Collins MJ, Riley MS, Child AM, Turner-Walker G (1995). A basic mathematical simulation of the chemical degradation of ancient collagen. J. Archaeol. Sci..

[21] France CAM, Thomas DB, Doney CR, Madden O (2014). FT-Raman spectroscopy as a method for screening collagen diagenesis in bone. J. Archaeol. Sci..

[22] Chadefaux C, Le Hô A-S, Bellot-Gurlet L, Reiche I (2009). Curve-fitting Micro-ATR-FTIR studies of the amide I and II bands of type I collagen in archaeological bone materials. E-Preserv. Sci. Morana RTD.

[23] Sponheimer M (2019). Saving old bones: A non-destructive method for bone collagen prescreening. Sci. Rep..

[24] Goldenberg L, Regev L, Mintz E, Boaretto E (2017). Dating reassembled collagen from fossil bones. Radiocarbon.

[25] Yizhaq M (2005). Quality controlled radiocarbon dating of bones and charcoal from the early pre-pottery neolithic B (PPNB) of Motza (Israel). Radiocarbon.

[26] Baker MJ (2014). Using Fourier transform IR spectroscopy to analyze biological materials. Nat. Protoc..

[27] Belbachir K, Noreen R, Gouspillou G, Petibois C (2009). Collagen types analysis and differentiation by FTIR spectroscopy. Anal. Bioanal. Chem..

[28] de Campos Vidal B, Mello MLS (2011). Collagen type I amide I band infrared spectroscopy. Micron.

[29] Figueiredo, M., Gamelas, J. & Martins, A. In *Infrared Spectroscopy-Life and Biomedical Sciences* (ed Theophile, T.) (InTech, 2012).

[30] Hanifi A, McCarthy H, Roberts S, Pleshko N (2013). Fourier transform infrared imaging and infrared fiber optic probe spectroscopy identify collagen type in connective tissues. PLoS ONE.

[31] Kong J, Yu S (2007). Fourier transform infrared spectroscopic analysis of protein secondary structures. Acta Biochim. Biophys. Sin..

[32] Stani C, Vaccari L, Mitri E, Birarda G (2020). FTIR investigation of the secondary structure of type I collagen: New insight into the amide III band. Spectrochim. Acta Part A Mol. Biomol. Spectrosc..

[33] Ramachandran G, Kartha G (1954). Structure of collagen. Nature.

[34] Ramachandran G, Kartha G (1955). Structure of collagen. Nature.

[35] Rich A, Crick F (1961). The molecular structure of collagen. J. Mol. Biol..

[36] Egli J, Schnitzer T, Dietschreit JC, Ochsenfeld C, Wennemers H (2019). Why proline? Influence of ring-size on the collagen triple helix. Org. Lett..

[37] Barth A (2007). Infrared spectroscopy of proteins. Biochim. Biophys. Acta Bioenergetics.

[38] Surovell TA, Stiner MC (2001). Standardizing infra-red measures of bone mineral crystallinity: An experimental approach. J. Archaeol. Sci..

[39] Garvie-Lok SJ, Varney TL, Katzenberg MA (2004). Preparation of bone carbonate for stable isotope analysis: The effects of treatment time and acid concentration. J. Archaeol. Sci..

[40] Hollund HI, Ariese F, Fernandes R, Jans MME, Kars H (2013). Testing an alternative high-throughput tool for investigating bone diagenesis: FTIR in attenuated total reflection (ATR) mode. Archaeometry.

[41] Berna F, Matthews A, Weiner S (2004). Solubilities of bone mineral from archaeological sites: The recrystallization window. J. Archaeol. Sci..

[42] Lebon M, Reiche I, Frohlich F, Bahain JJ, Falgueres C (2008). Characterization of archaeological burnt bones: Contribution of a new analytical protocol based on derivative FTIR spectroscopy and curve fitting of the nu1nu3 PO_4_ domain. Anal. Bioanal. Chem..

[43] Thompson TJU, Gauthier M, Islam M (2009). The application of a new method of Fourier Transform Infrared Spectroscopy to the analysis of burned bone. J. Archaeol. Sci..

[44] Lebon M (2010). New parameters for the characterization of diagenetic alterations and heat-induced changes of fossil bone mineral using Fourier transform infrared spectrometry. J. Archaeol. Sci..

[45] Dal Sasso G (2016). Bone diagenesis variability among multiple burial phases at Al Khiday (Sudan) investigated by ATR-FTIR spectroscopy. Palaeogeogr. Palaeoclimatol. Palaeoecol..

[46] Toffolo MB, Brink JS, Berna F (2015). Bone diagenesis at the Florisbad spring site, Free State Province (South Africa): Implications for the taphonomy of the Middle and Late Pleistocene faunal assemblages. J. Archaeol. Sci. Rep..

[47] Lebon M, Reiche I, Gallet X, Bellot-Gurlet L, Zazzo A (2016). Rapid quantification of bone collagen content by ATR-FTIR spectroscopy. Radiocarbon.

[48] Pestle WJ (2015). Hand-held Raman spectroscopy as a pre-screening tool for archaeological bone. J. Archaeol. Sci..

[49] Madden O, Chan DMW, Dundon M, France CAM (2018). Quantifying collagen quality in archaeological bone: Improving data accuracy with benchtop and handheld Raman spectrometers. J. Archaeol. Sci. Rep..

[50] Dal Sasso G, Angelini I, Maritan L, Artioli G (2018). Raman hyperspectral imaging as an effective and highly informative tool to study the diagenetic alteration of fossil bones. Talanta.

[51] López-Costas O, Müldner G (2016). Fringes of the empire: Diet and cultural change at the Roman to post-Roman transition in NW Iberia. Am. J. Phys. Anthropol..

[52] López-Costas, O. *Antropología de los restos óseos humanos de Galicia: estudio de la población romano y medieval gallega. Doctoral thesis*, University of Granada, (2012).

[53] Petibois C, Gouspillou G, Wehbe K, Delage J-P, Déléris G (2006). Analysis of type I and IV collagens by FT-IR spectroscopy and imaging for a molecular investigation of skeletal muscle connective tissue. Anal. Bioanal. Chem..

[54] Haris PI, Severcan F (1999). FTIR spectroscopic characterization of protein structure in aqueous and non-aqueous media. J. Mol. Catal. B Enzym..

[55] Goormaghtigh E, Ruysschaert J-M, Raussens V (2006). Evaluation of the information content in infrared spectra for protein secondary structure determination. Biophys. J ..

[56] Paschalis EP (2001). Spectroscopic characterization of collagen cross-links in bone. J. Bone Miner. Res..

[57] D'Elia M (2007). Evaluation of possible contamination sources in the 14C analysis of bone samples by FTIR spectroscopy. Radiocarbon.

[58] Karkanas P, Bar-Yosef O, Goldberg P, Weiner S (2000). Diagenesis in prehistoric caves: The use of minerals that form in situ to assess the completeness of the archaeological record. J. Archaeol. Sci..

[59] López-Costas O, Lantes-Suárez Ó, Martínez Cortizas A (2016). Chemical compositional changes in archaeological human bones due to diagenesis: Type of bone vs soil environment. J. Archaeol. Sci..

[60] Trueman CN, Privat K, Field J (2008). Why do crystallinity values fail to predict the extent of diagenetic alteration of bone mineral?. Palaeogeogr. Palaeoclimatol. Palaeoecol..

[61] Trueman CNG, Behrensmeyer AK, Tuross N, Weiner S (2004). Mineralogical and compositional changes in bones exposed on soil surfaces in Amboseli National Park, Kenya: Diagenetic mechanisms and the role of sediment pore fluids. J. Archaeol. Sci..

[62] Salesse K (2014). Variability of bone preservation in a confined environment: The case of the catacomb of Sts Peter and Marcellinus (Rome, Italy). Palaeogeogr. Palaeoclimatol. Palaeoecol..

[63] Weiner S (2010). Microarchaeology: Beyond the Visible Archaeological Record.

[64] Pate FD, Hutton JT, Norrish K (1989). Ionic exchange between soil solution and bone: Toward a predictive model. Appl. Geochem..

[65] Nielsen-Marsh CM, Hedges REM (2000). Patterns of diagenesis in bone I: The effects of site environments. J. Archaeol. Sci..

[66] Weiner S, Bar-Yosef O (1990). States of preservation of bones from prehistoric sites in the Near East: A survey. J. Archaeol. Sci..

[67] Weiner S, Goldberg P, Bar-Yosef O (1993). Bone preservation in Kebara cave, Israel using on-site Fourier transform infrared spectrometry. J. Archaeol. Sci..

[68] Weiner S, Goldberg P, Bar-Yosef O (2002). Three-dimensional distribution of minerals in the sediments of Hayonim Cave, Israel: Diagenetic processes and archaeological implications. J. Archaeol. Sci..

[69] Jans MME, Nielsen-Marsh CM, Smith CI, Collins MJ, Kars H (2004). Characterisation of microbial attack on archaeological bone. J. Archaeol. Sci..

[70] Ambrose SH (1990). Preparation and characterization of bone and tooth collagen for isotopic analysis. J. Archaeol. Sci..

[71] López-Costas O, Müldner G, Martínez Cortizas A (2015). Diet and lifestyle in Bronze Age Northwest Spain: The collective burial of Cova do Santo. J. Archaeol. Sci..

[72] Lopez-Costas O (2015). Taphonomy and burial context of the Roman/post-Roman funerary areas (2nd to 6th centuries AD) of A Lanzada, NW Spain. Estudos do Quaternário, APEQ.

[73] Collins MJ, Galley P (1998). Towards an optimal method of archaeological collagen extraction: The influence of pH and grinding. Ancient Biomolecules.

[74] Boskey A, Camacho NP (2007). FT-IR imaging of native and tissue-engineered bone and cartilage. Biomaterials.

[75] Kim M, Bi X, Horton W, Spencer R, Camacho N (2005). Fourier transform infrared imaging spectroscopic analysis of tissue engineered cartilage: Histologic and biochemical correlations. J. Biomed. Opt..

[76] Heinly, J. H., Guerin, H. L., Auerbach, J. D., Siskey, R. L. & Villarraga, M. L. In *56th Annual Meeting of the Orthopaedic Research Society* Poster No. 1466 (2010.).

[77] Mark H, Workman J (2003). Chemometrics: Derivatives in spectroscopy, Part I-the behavior of the derivative. Spectrosc. Eugene.

[78] Rieppo L (2012). Application of second derivative spectroscopy for increasing molecular specificity of fourier transform infrared spectroscopic imaging of articular cartilage. Osteoarthr. Cartil..

[79] Ami, D., Mereghetti, P. & Doglia, S. M. In *Multivariate Analysis in Management, Engineering and the Sciences* (eds de Freitas, L. V. & de Freitas, A. P. B. R.) https://www.intechopen.com/books/multivariate-analysis-in-management-engineering-and-the-sciences/multivariate-analysis-for-fourier-transform-infrared-spectra-of-complex-biological-systems-and-proce (Intech Open, 2013).

[80] Saarakkala, S., Rieppo, L., Rieppo, J. & Jurvelin, J. In *Microscopy: Science, Technology, Applications and Education* Vol. 1 (eds Méndez-Vilas, A. & Díaz, J.) 403–414 (Formatex, 2010).

[81] Smith, B. C. (CRC Press, Boca Raton, 2011).

[82] Eriksson L, Johansson E, Kettaneh-Wold N, Wold S (1999). Introduction to Multi- and Megavariate Data Analysis using Projection Methods (PCA & PLS).

[83] Garson, G. D. In *Blue Book Series* (Statistical Associates Publishers, Asheboro, 2016).

[84] SmartPLS 3 (SmartPLS GmbH, Boenningstedt, 2015).

