# Supplementary material for: Linking structural and compositional changes in archaeological human bone collagen: an FTIR-ATR approach
Source: Sci Rep. 2020 Oct 21;10:17888. doi: 10.1038/s41598-020-74993-y (PMC7578014; doi:10.1038/s41598-020-74993-y)
Supplement: Supplementary file 1 — Supplementary Information. [file 41598_2020_74993_MOESM1_ESM.docx]

Supplementary information

Linking structural and compositional changes in archaeological human bone collagen: an FTIR-ATR approach.

Antonio Martínez Cortizas and Olalla López-Costas

SI_Figure 1. Location and synthetic information of the studied necropoleis from NW Iberia. For the codes of the necropoleis see SI_Table 1. Map modified from Kaal et al.[^1^](#_ENREF_1) using QGIS (3.4.10-Madeira, GNU public licence <https://qgis.org/es/site/>).


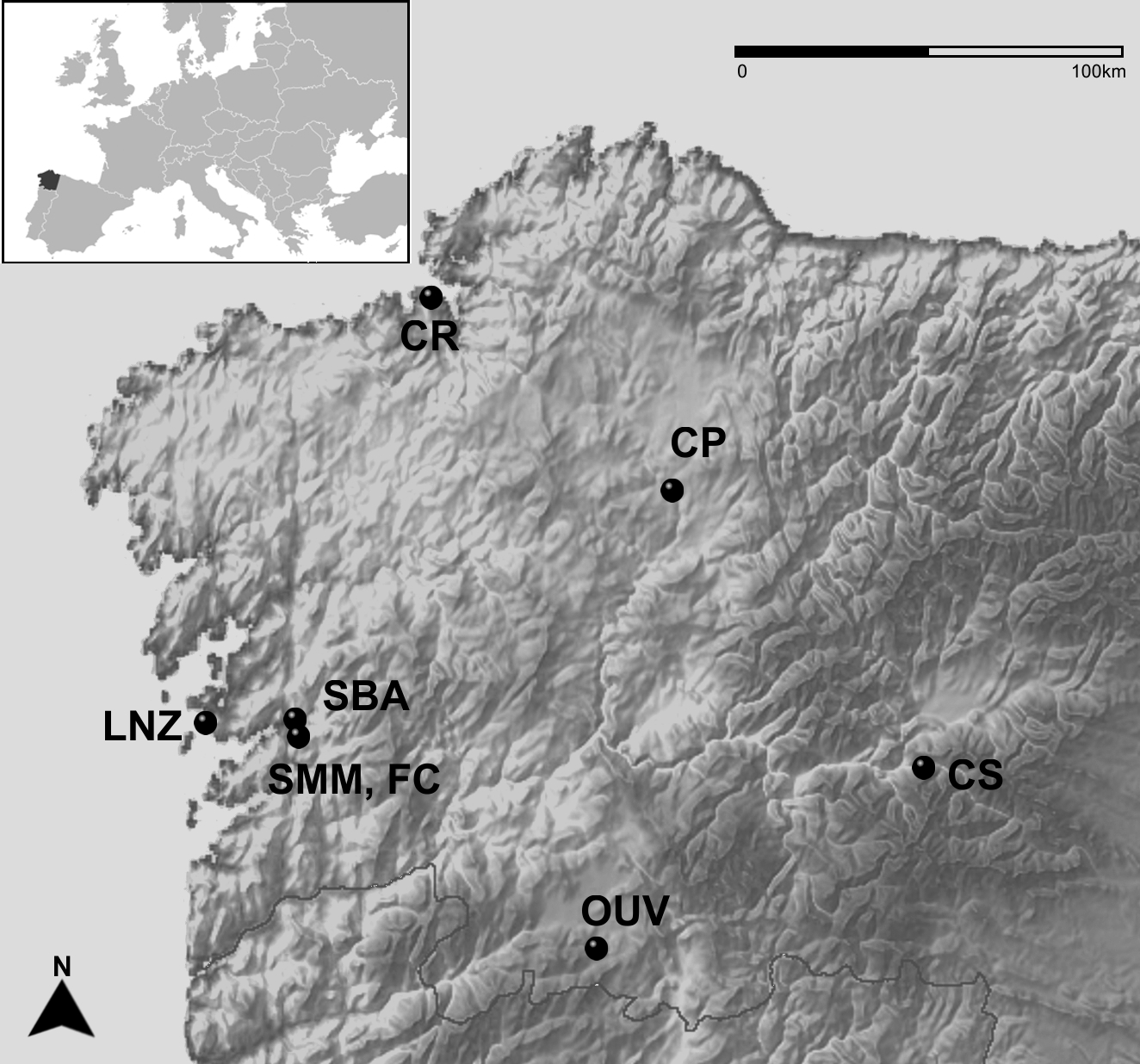


SI_Figure 2. A) Standardized spectra and B) second derivative spectra of the extracted collagen samples.


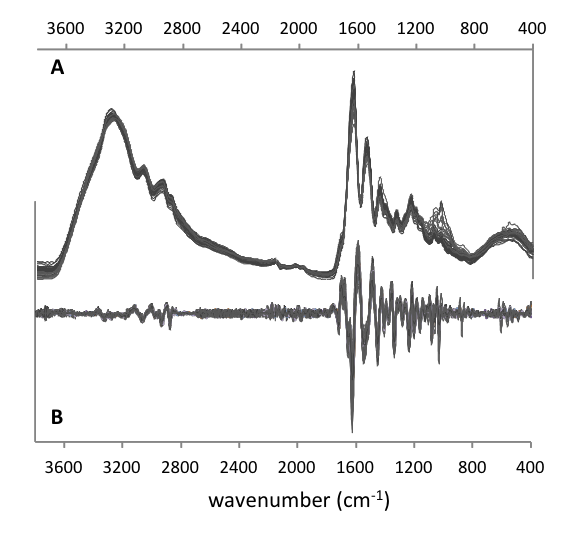


SI_Figure 3. Total effects of the initial PLS-SEM model relating collagen composition (predictor LVs; as reflected by the main FTIR absorptions) to collagen quality and collagen isotopic composition. It can be seen that the backbone lipids LV has an almost negligible total effect on collagen quality and is highly correlated to the amides LV.


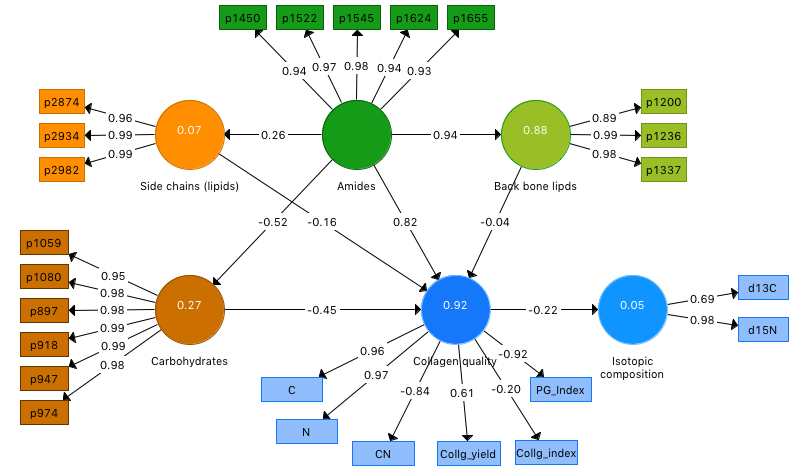


SI_Table 1. Summary data of the necropoleis and samples analysed in this study. All individuals were over 10 years old and belong to primary inhumation burials except CS (comingled remains). Ad= adults >20 years old; Sub=subadults ≤19 years old / c. century

| Site (town/province) | Code | Age | Burial environment | n | ♂/♀ | Ad/Sub |
| --- | --- | --- | --- | --- | --- | --- |
| Cova do Santo  (Pardollán/Ourense) | CS | 19^th^-16t^h^ BC | Limestone cave | 5 | 1/3 | 5/- |
| Lanzada  (Noalla/Pontevedra) | LNZ | 1^st^-6^th^ AD | Paleodune (sands) and loamy acidic soil | 10 | 5/4 | 9/1 |
| Rúa Real  (Coruña/Coruña) | CR | 5^th^-7^th^ AD | Paleodune (sands) | 5 | 1/1 | 5/- |
| Ouvigo  (Os Blancos/Ourense) | OUV | 4^th^-13^th^ AD | Silty-loam acidic soil | 7 | 3/4 | 6/1 |
| Capela do Pilar  (Lugo/Lugo) | CP | 11^th^-14^th^ AD | Silty-clay acidic soil | 3 | 3/- | 3/- |
| San Bartolomé church  (Pontevedra/Pontevedra) | SBA | 13^th^-15^th^ AD | Sandy-loam acidic soil | 6 | 3/3 | 6/- |
| Santa María church (Pontevedra/Pontevedra) | SMM | 13^th^-17^th^ AD | Sandy-loam acidic soil | 7 | 4/2 | 6/1 |
| Santa María mass grave  (Pontevedra/Pontevedra) | FC | 17^th^ or 18^th^ AD | Sandy acidic soil | 7 | 2/3 | 5/2 |

SI_Table 2. Band assignments for the relevant FTIR absorptions of extracted collagen samples. It includes stretching vibrations (ν), in plane bending vibrations (δ) and out of plane bending vibrations (γ) for the different molecular bonds. Am: amides; Cbh: carbohydrates; Lip: lipids.

| **WN/ (cm^-1^)** | **Bond νibration** | **Assignation** | **References** |
| --- | --- | --- | --- |
| **897** | ν(C-O) ν(C-H) δ(C-O-H) δ(C-O-C) | Cbh | 2, 8 |
| **918** | ν(C-O) ν(C-H) δ(C-O-H) δ(C-O-C) | Cbh | 2, 8 |
| **947** | ν(C-O) ν(C-H) δ(C-O-H) δ(C-O-C) | Cbh | 2, 8 |
| **974** | ν(C-O) ν(C-H) δ(C-O-H) δ(C-O-C) | Cbh | 2, 8 |
| **1030** | ν(C-O) | Cbh | 9, 15, 20 |
| **1059** | ν(C-O) | Cbh | 2, 8, 9, 15, 20 |
| **1080** | ν(C-O) | Cbh | 2, 8, 9, 12, 15, 20 |
| **1121** | ν(C-O) ν(C-O-C) | Cbh | 2, 8, 15 |
| **1200** | δ(N-H) ν(C-N) | AmIII | 3, 19 |
| **1236** | δ(N-H) ν(C-N) | AmIII | 4, 5, 7, 9, 12-16 |
| **1337** | δ(CH2) δ(N-H) ν(C-N) | AmII.AmIII | 2, 12, 19 |
| **1450** | δ(CH2) δ(CH_3_) | Am. Lip. Cbh | 2-4, 7, 15, 12, 19, 20 |
| **1522** | δ(N-H) ν(C-N) | AmII | 13, 2, 9, 11, 19, 20 |
| **1545** | δ(N-H) ν(C-N) | AmII | 4-6, 8, 9, 13-16, 19, 20 |
| **1624** | ν(C=O) | AmI | 3, 5-7, 11, 13, 16-19 |
| **1655** | ν(C=O) | AmI | 4-6, 8, 9-10, 13-16, 19, 20 |
| **1690** | ν(C=O) | AmI | 3, 9, 20, 11-13, 16, 17, 19 |
| **1719** | ν(C=O) | AmI | 9, 11 |
| **2874** | ν(C-H) ν(CH_3_) | AmB. Lip | 2, 7, 8, 12, 19 |
| **2930** | ν(C-H) ν(CH_2_) | AmB. Lip | 2, 6-8, 12, 19 |
| **2982** | ν(C-H) | AmB. Lip | 2, 7, 8, 12, 19 |
| **3070** | ν(C-H) ν(N-H) | AmA. AmB. Lip | 2, 8, 8, 12, 13, 19 |
| **3320** | ν(N-H) | AmA | 5, 13, 19 |
| **3458** | ν(N-H) | AmA | 15 |

SI_Table 3. Analysis of variance for the grouping factors: archaeological site (Site), burial period (Period), age-at-death, sex and geochemical environment (Burial). The F statistics and probability (P) are provided. Sites: LZ: A Lanzada, CR: Rua Real, OU: Ouvigo, SB: San Bartolomé, SM: Santa María la Mayor, FC: mass grave. Period: Ba, Bronze age; Rom, Roman; pRom, post-Roman; Med-Mod, Medieval to Modern. Age-at-death, in years. Letters indicate homogenous groups (Tukey test, P<0.05).

| **Site** | **F** | **P** | **CS** | **LZ** | **CR** | **OU** | **SB** | | **CP** | **SM** | **FC** | |
| --- | --- | --- | --- | --- | --- | --- | --- | --- | --- | --- | --- | --- |
| LVst | 1.93 | 0.09 | 0.85b | 0.19ab | 0.52b | -0.31ab | -0.05ab | | -1.35a | -0.03ab | -0.29ab | |
| LVcb | 2.60 | 0.03 | -0.64a | 0.26ab | -0.64a | 0.56ab | -0.48a | | 1.31b | 0.84ab | -0.42a | |
| LVsc | 1.16 | 0.34 | -0.34a | 0.19a | 0.14a | 0.48a | -0.71a | | 0.63a | 0.17a | -0.40a | |
| LVcq | 3.16 | <0.01 | 1.10c | -0.25ab | 0.64bc | -0.61ab | 0.35ab | | -1.31a | -0.07ab | 0.04ab | |
|  |  |  |  |  |  |  |  | |  |  |  | |
| **Period** | **F** | **P** | **Ba** | **Rom** | **pRom** | **Med-Mod** |  | |  |  |  | |
| LVst | 2.04 | 0.12 | 0.86a | 0.19a | 0.04a | -0.29a |  | |  |  |  | |
| LVcb | 0.89 | 0.45 | -0.64a | 0.26a | 0.07a | -0.01a |  | |  |  |  | |
| LVsc | 1.04 | 0.38 | -0.34a | 0.19a | 0.34a | -0.19a |  | |  |  |  | |
| LVcq | 2.47 | 0.07 | 1.10b | -0.25a | -0.09ab | -0.08ab |  | |  |  |  | |
|  |  |  |  |  |  |  |  | |  |  |  | |
| **Age-at-death** | **F** | **P** | **<19** | **20-39** | **40-59** | **>60** |  |  | |  | |  |
| LVst | 0.16 | 0.92 | -0.45a | -0.02a | -0.28a | -0.10a |  |  | |  | |  |
| LVcb | 0.98 | 0.42 | 0.48a | -0.39a | 0.20a | 0.16a |  |  | |  | |  |
| LVsc | 0.13 | 0.99 | -0.20a | -0.11a | -0.08a | -0.15a |  |  | |  | |  |
| LVcq | 0.17 | 0.91 | -0.26a | 0.08a | -0.19a | -0.03a |  |  | |  | |  |
|  |  |  |  |  |  |  |  | |  |  |  | |
| **Sex** | **F** | **P** | **Male** | **Female** |  | **Burial** | **F** | | **P** | **Acidic** | **Alkaline** | |
| LVst | 2.12 | 0.15 | -0.20 | 0.24 |  | LVst | 2.87 | | 0.09 | -0.10 | 0.54 | |
| LVcb | 2.28 | 0.10 | 0.30 | -0.23 |  | LVpg | 3.02 | | 0.09 | 0.11 | -0.54 | |
| LVsc | 0.07 | 0.79 | 0.01 | 0.09 |  | LVsc | 0.79 | | 0.38 | 0.06 | -0.29 | |
| LVcq | 1.39 | 0.25 | -0.21 | 0.16 |  | LVcq | 4.79 | | 0.03 | -0.13 | 0.69 | |

References for SI fig 1 and SI_Table 1: [^2-20^](#_ENREF_2)

1 Kaal, J., López-Costas, O. & Martínez Cortizas, A. Diagenetic effects on pyrolysis fingerprints of extracted collagen in archaeological human bones from NW Spain, as determined by pyrolysis-GC-MS. *Journal of archaeological science* **65**, 1-10, doi:<http://dx.doi.org/10.1016/j.jas.2015.11.001> (2016).

2 Ami, D., Mereghetti, P. & Doglia, S. M. in *Multivariate Analysis in Management, Engineering and the Sciences* (eds Leandro Valim de Freitas & Ana Paula Barbosa Rodrigues de Freitas) https://[www.intechopen.com/books/multivariate-analysis-in-management-engineering-and-the-sciences/multivariate-analysis-for-fourier-transform-infrared-spectra-of-complex-biological-systems-and-proce](http://www.intechopen.com/books/multivariate-analysis-in-management-engineering-and-the-sciences/multivariate-analysis-for-fourier-transform-infrared-spectra-of-complex-biological-systems-and-proce) (Intech Open, 2013).

3 Belbachir, K., Noreen, R., Gouspillou, G. & Petibois, C. Collagen types analysis and differentiation by FTIR spectroscopy. *Anal Bioanal Chem* **395**, 829-837, doi:10.1007/s00216-009-3019-y (2009).

4 Chadefaux, C., Le Hô, A.-S., Bellot-Gurlet, L. & Reiche, I. Curve-fitting Micro-ATR-FTIR studies of the amide I and II bands of type I collagen in archaeological bone materials. *E-Preservation Science Morana RTD* **6**, 129-137 (2009).

5 de Campos Vidal, B. & Mello, M. L. S. Collagen type I amide I band infrared spectroscopy. *Micron* **42**, 283-289, doi:https://doi.org/10.1016/j.micron.2010.09.010 (2011).

6 Figueiredo, M., Gamelas, J. & Martins, A. in *Infrared Spectroscopy-Life and Biomedical Sciences* (ed Theophile Theophanides) (InTech, 2012).

7 France, C. A. M., Thomas, D. B., Doney, C. R. & Madden, O. FT-Raman spectroscopy as a method for screening collagen diagenesis in bone. *Journal of archaeological science* **42**, 346-355, doi:<http://dx.doi.org/10.1016/j.jas.2013.11.020> (2014).

8 Ede, S., Hafner, L., Frost, R. & Will, G. Inactivation and structural changes of E. cloacae and B. subtilis endospores during IR laser water treatment. *Hydrology: Current Research* **3** (2012).

9 Hanifi, A., McCarthy, H., Roberts, S. & Pleshko, N. Fourier Transform Infrared Imaging and Infrared Fiber Optic Probe Spectroscopy Identify Collagen Type in Connective Tissues. *PLoS ONE* **8**, e64822, doi:10.1371/journal.pone.0064822 (2013).

10 Heredia, A. *et al.* Thermal, infrared spectroscopy and molecular modeling characterization of bone: An insight in the apatite-collagen type I interaction. *Advances in Biological Chemistry* **Vol.03No.02**, 9, doi:10.4236/abc.2013.32027 (2013).

11 Hu, X. *et al.* Stability of Silk and Collagen Protein Materials in Space. *Scientific Reports* **3**, 3428, doi:10.1038/srep03428 (2013).

12 Jonker, J.-L. *et al.* The chemistry of stalked barnacle adhesive (*Lepas anatifera*). *Interface Focus* **5**, 20140062, doi:doi:10.1098/rsfs.2014.0062 (2015).

13 Kong, J. & Yu, S. Fourier Transform Infrared Spectroscopic Analysis of Protein Secondary Structures. *Acta Biochimica et Biophysica Sinica* **39**, 549-559, doi:10.1111/j.1745-7270.2007.00320.x (2007).

14 Mitra, T., Sailakshmi, G., Gnanamani, A. & Mandal, A. B. Studies on Cross-linking of succinic acid with chitosan/collagen. *Materials Research* **16**, 755-765 (2013).

15 Muyonga, J. H., Cole, C. G. B. & Duodu, K. G. Fourier transform infrared (FTIR) spectroscopic study of acid soluble collagen and gelatin from skins and bones of young and adult Nile perch (Lates niloticus). *Food Chemistry* **86**, 325-332, doi:https://doi.org/10.1016/j.foodchem.2003.09.038 (2004).

16 Nagai, T. Characterization of Acid-Soluble Collagen from Skins of Surf Smelt (Hypomesus pretiosus japonicus Brevoort). *Food and Nutrition Sciences* **Vol.01No.02**, 8, doi:10.4236/fns.2010.12010 (2010).

17 Odlyha, M., Theodorakopoulos, C., de Groot, J., Bozec, L. & Horton, M. Fourier transform infra-red spectroscopy (ATR/FTIR) and scanning probe microscopy of parchment. *e-Preservation Science* **6**, 138-144 (2009).

18 Pestle, W. J., Ahmad, F., Vesper, B. J., Cordell, G. A. & Colvard, M. D. Ancient bone collagen assessment by hand-held vibrational spectroscopy. *Journal of archaeological science* **42**, 381-389, doi:<http://dx.doi.org/10.1016/j.jas.2013.11.014> (2014).

19 Rahman, M. A. & Halfar, J. First evidence of chitin in calcified coralline algae: new insights into the calcification process of Clathromorphum compactum. *Scientific Reports* **4**, 6162, doi:10.1038/srep06162 (2014).

20 Rieppo, L. *et al.* Application of second derivative spectroscopy for increasing molecular specificity of fourier transform infrared spectroscopic imaging of articular cartilage. *Osteoarthritis and Cartilage* **20**, 451-459, doi:https://doi.org/10.1016/j.joca.2012.01.010 (2012).
